# Supplementary material for: Failure of DNA double-strand break repair by tau mediates Alzheimer’s disease pathology in vitro
Source: Commun Biol. 2022 Apr 13;5:358. doi: 10.1038/s42003-022-03312-0 (PMC9008043; doi:10.1038/s42003-022-03312-0)

Suppl. Table 1. Characteristics of human brain samples.

	Case	Age at death(years)	Sex	Clinical diagnosis	NFT stage	CERAD
Control	1	74	F	Suffocation	0	B
	2	68	M	Rheumatoid arthritis	0	0
	3	70	F	Cerebral hemorrhage	0	0
	4	68	F	Breast cancer	0	0
	5	74	M	Lung cancer	III	B
	6	94	F	Disseminated intravascular coagulation	II	0
	7	92	M	Prostate cancer	III	0
	8	86	M	Intracranial hemorrhage	III	0
AD	1	89	F	Alzheimer disease	V	C
	2	93	F	Alzheimer disease	V	C
	3	85	M	Alzheimer disease	VI	C
	4	77	F	Alzheimer disease	V	C
	5	85	M	Alzheimer disease	IV	C
	6	76	F	Alzheimer disease	VI	C
	7	67	F	Alzheimer disease	VI	C

Clinical and histopathological information of the human brain samples used in this study. We analyzed five brains from patients with neuropathology-confirmed AD and five brains from non-neurodegenerative disease control subjects. NFT, neurofibrillary tangle; CERAD, Consortium to Establish a Registry for Alzheimer's Disease.

Suppl.Fig.1

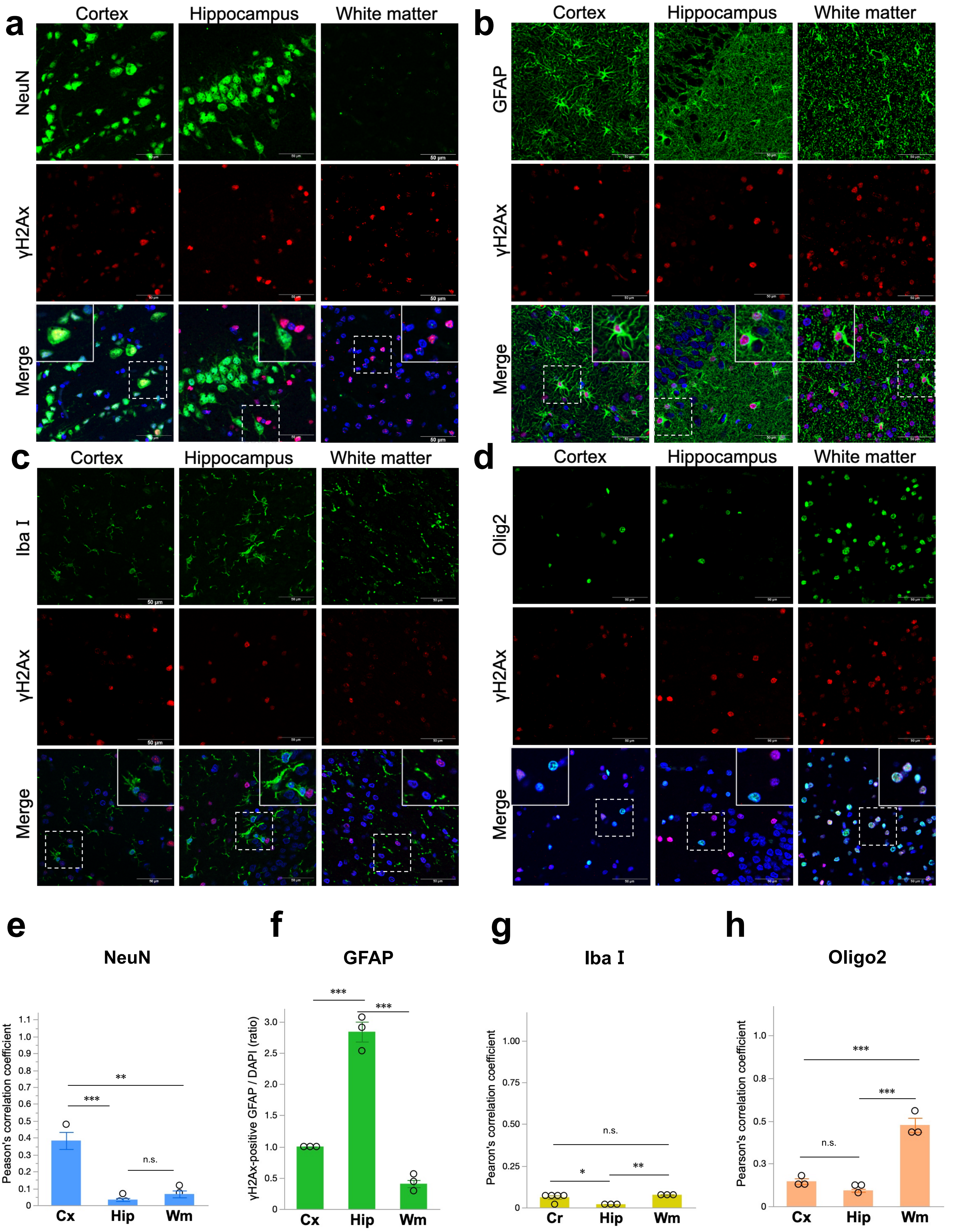
a
Cortex
Hippocampus
White matter
NeuN
γH2Ax
Merge
b
Cortex
Hippocampus
White matter
GFAP
γH2Ax
Merge
c
Cortex
Hippocampus
White matter
Iba I
γH2Ax
Merge
d
Cortex
Hippocampus
White matter
Olig2
γH2Ax
Merge
e
NeuN
Peason's correlation coefficient
Cx
Hip
Wm
**
***
n.s.
f
GFAP
γH2Ax-positive GFAP / DAPI (ratio)
Cx
Hip
Wm
***
***
g
Iba I
Pearon's correlation coefficient
Cr
Hip
Wm
n.s.
*
**
h
Oligo2
Pearson's correlation coefficient
Cx
Hip
Wm
***
***
n.s.

Suppl.Fig.1

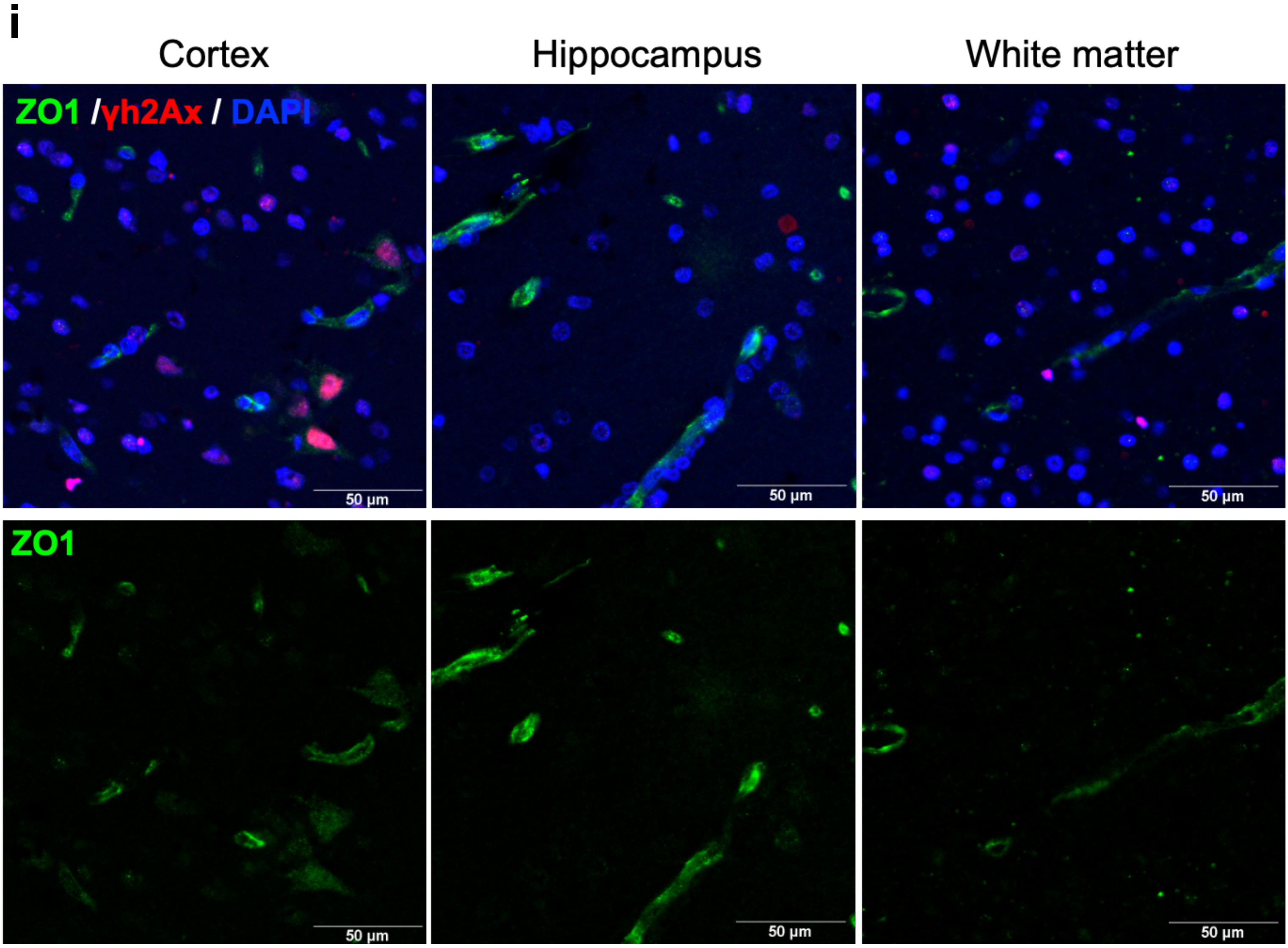

Suppl. Fig. 1 Cell-type predominancy of DSB in the cortex , hippocampus and white matter of AD brains.
Immunofluorescence images of γH2Ax (red) with **(a)** NeuN (green) of neuronal marker , **(b)** GFAP (green) of astrocyte marker, **(c)** Iba1 (green) of microglia marker, **(d)** Olig2 (green) of oligodendrocyte marker and **(i)** ZO1(green) of endothelial cell marker. White boxes are zoomed images of dash boxes. Scale bar = 50 µm. **(e)** Pearson's correlation coefficient values showing predominant overlay between NeuN and γH2Ax in the cortex (n=3, $^{**}p$=0.001, $^{***}p$=0.0006), **(f)** between; γH2Ax and GFAP in hippocompus (n=3, Cx vs. Hip; $^{***}p$=0.0003, Hip vs. Wm; $^{***}p < 0.0001$), **(g)** between; γH2Ax and Iba1 (n=3, $^{*}p$=0.0241, $^{**}p$=0.0091), and **(h)**between**;** γH2Ax and Olig2 in the white matter (n=3, Cx vs. Wm; $^{***}p$=0.0003, Hip vs. Wm; $^{***}p = 0.0001$). n: number of images examined; Statistical significance was determined by two-way ANOVA, followed by a Tukey test.

Suppl.Fig.2

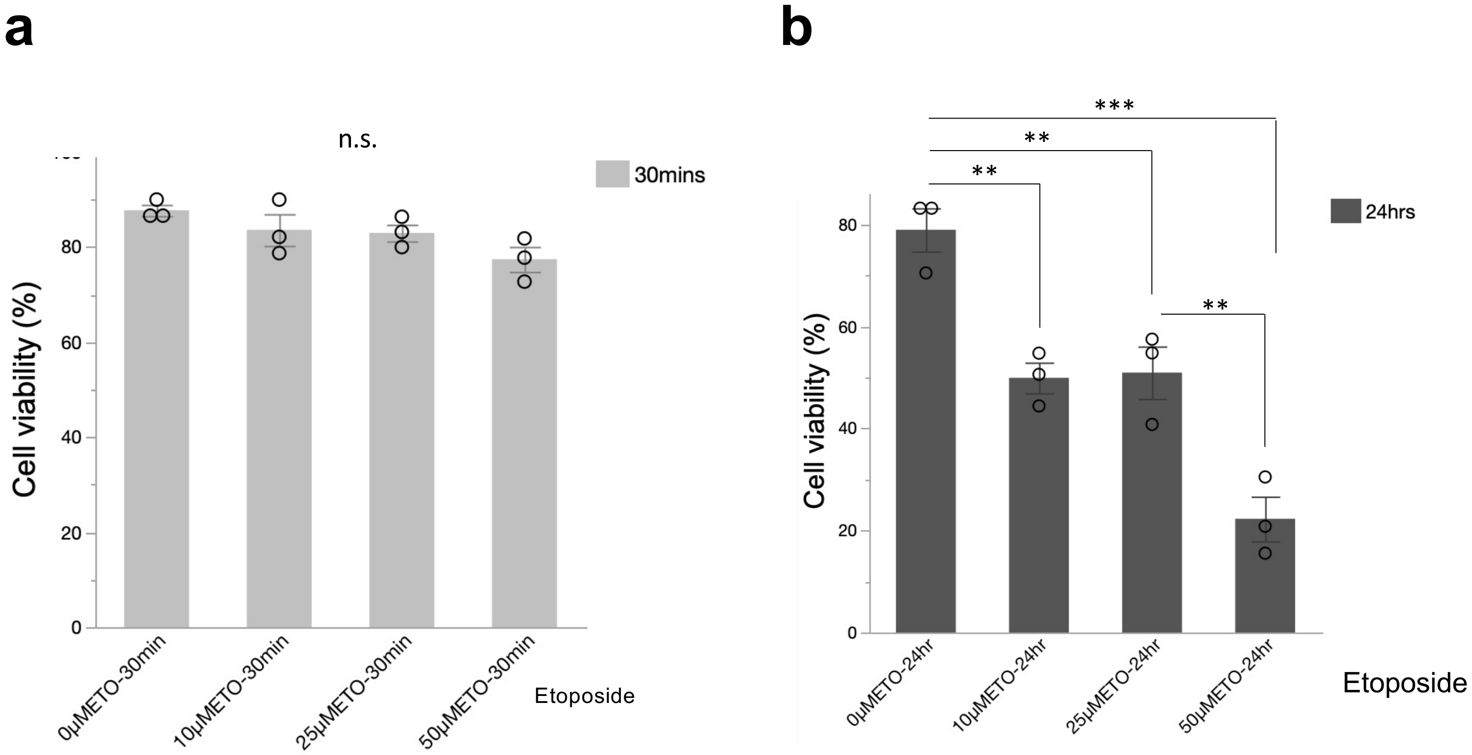

Suppl. Fig. 2 Primary mouse cortical neurons viability assay after etoposide exposure
Primary mouse cortical neuron cultures (DIV 7) were treated by designated concentrations of etoposide for 30 mins **(a)** or 24 hrs **(b)** and incubated at room temperature for 10 mins in 0.4% Trypan Blue. Cells were fixed with 4% PFA for 15 mins, followed by counting live and dead cells. Cell death was not observed 30 mins after treatment **(a)**. 24 hrs after treatment, cell viability significantly decreased to about 50% with 10 and 25 µM treatment, and with 50 µM, it further decreased to about 25% **(b)**. Statistical significance was determined by one-way ANOVA, followed by a Tukey test. n=3, 0 µM vs 50 µM, $^{***}p<0.0001$; 0 µM vs 10 µM, $^{**}p=0.0058$; 25 µM vs 50 µM, $^{**}p=0.0062$; 0 µM vs 25 µM, $^{**}p=0.0071$; 10 µM vs 50 µM, $^{**}p=0.0077$.

Suppl.Fig.3

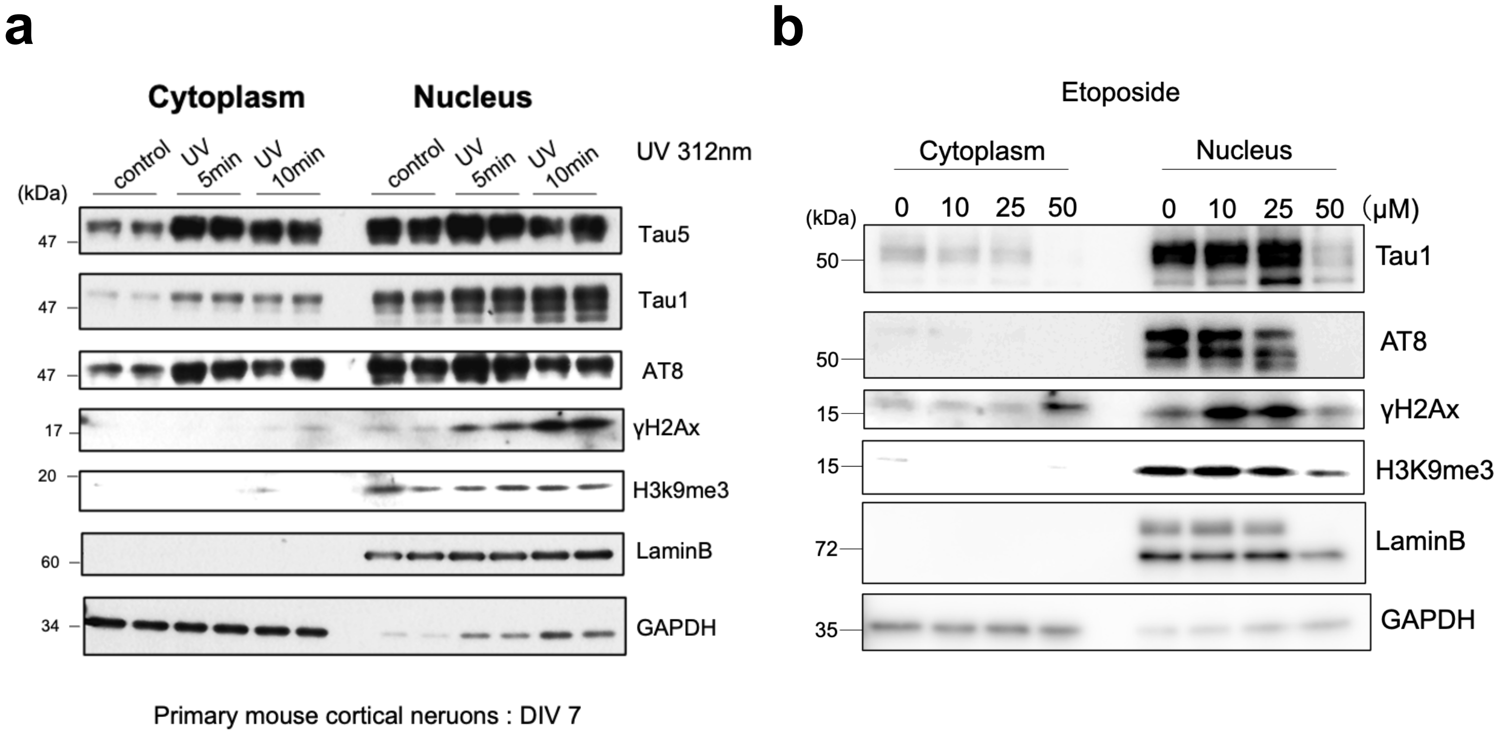

Suppl. Fig. 3 Increase of Tau in the nuclear fraction following DSB induction with UV exposure and etoposide treatment onto mature neurons.

(a) Primary mouse cortical neuron cultures (DIV 7) in 10 cm dish were exposed to UV light by the UV transilluminator (ATTO), separated into nuclear and cytoplasmic fractions, and were subjected to western blot analysis. H3K9me3, Heterochromatin marker; LaminB, nuclei marker; GAPDH, cytoplasm marker. (b) Primary mouse cortical neuron cultures (DIV 14) were treated with etoposide in a dose-dependent manner.

Suppl.Fig.4

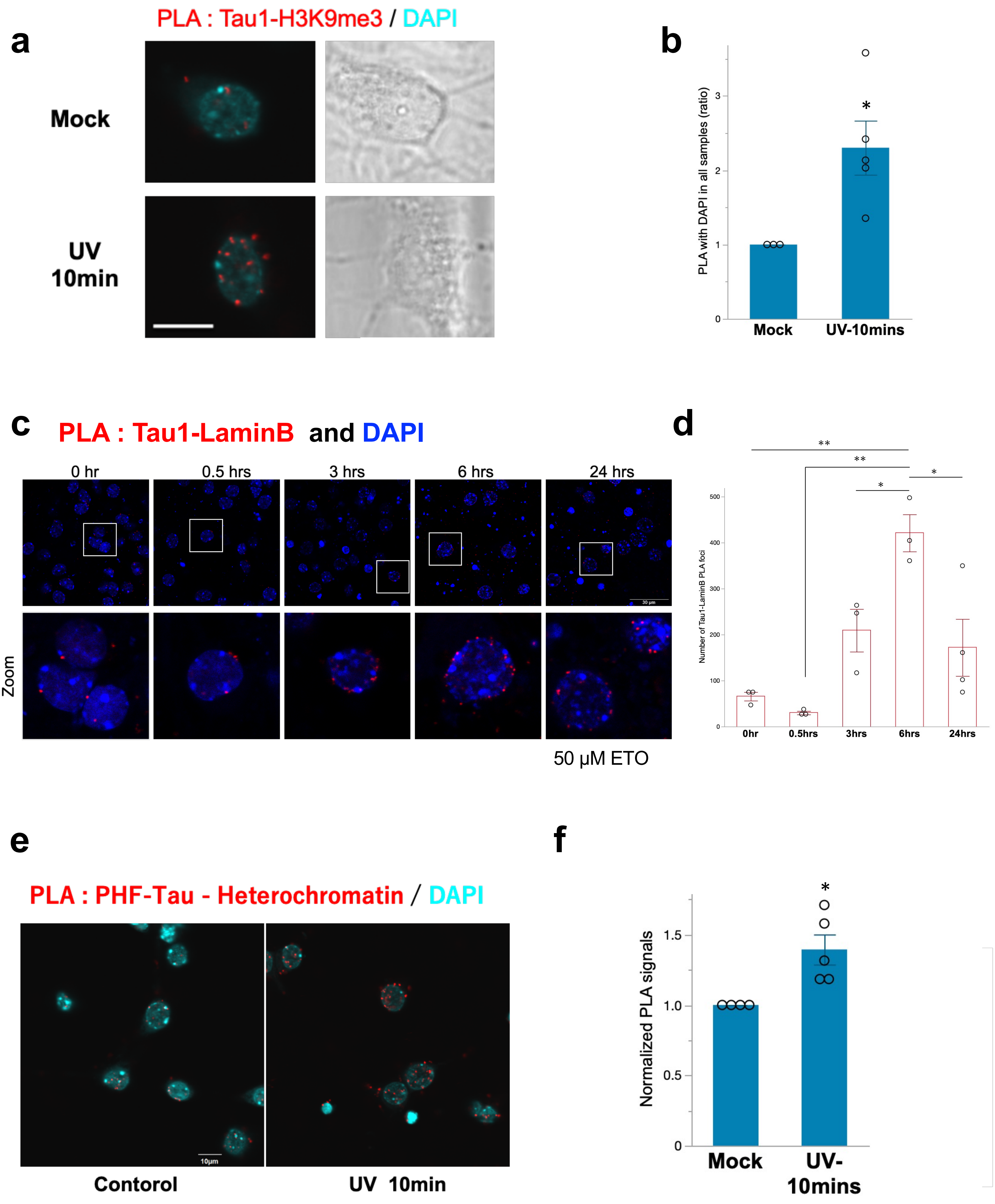
a
PLA : Tau1-H3K9me3 / DAPI
Mock
UV
10min
b
PLA with DAPI in all samples (ratio)
0
1
2
3
*
Mock
UV-10mins
c
PLA : Tau1-LaminB and DAPI
0 hr
0.5 hrs
3 hrs
6 hrs
24 hrs
Zoom
50 μM ETO
d
**
**
*
*
Number of Tau1-LaminB PLA foci
0
100
200
300
400
500
0hr
0.5hrs
3hrs
6hrs
24hrs
e
PLA : PHF-Tau - Heterochromatin / DAPI
10μm
Contorol
UV 10min
f
Normalized PLA signals
0
0.5
1.0
1.5
*
Mock
UV-
10mins

Suppl. Fig. 4 PLA assay for tau and nuclear membrane protein under DSB induction
(a) Left panels; PLA signals (red) for non-p-tau (Tau1) and heterochromatin marker (H3K9me3) with UV exposure for 10 mins in primary mouse cortical neuron cultures (DIV 7). Right panels; Phase-contrast images. Scale bar =10 µm. **(b)** Quantification of PLA signals for colocalized Tau1-H3K9me3 with DAPI ; n=3, UV ; n=5, $^{*}p$=0.0364, n: number of images examined; Student's T-test. **(c)** Upper panels; PLA signals (red) for Tau1 and LaminB at 0, 0.5, 3, 6 and 24 hrs after 50 µM etoposide treatment into primary mouse cortical neuron cultures (DIV 7). Scale bar =30 µm. Bottom panels; Magnified images of the white square box. **(d)** The number of PLA foci in primary mouse cortical neurons exposed to 50 µM etoposide for 0 hr (n=3), 0.5 hrs (n=3), 3 hrs (n=3), 6 hrs (n=3) and 24 hrs (n=4). n: number of images examined; The *p*-value was determined by two-way ANOVA, followed by a Tukey test. 0 hr vs 6 hrs., $^{**}p$=0.0014; 0.5 hrs vs 6 hrs., $^{**}p$=0.0006; 3hr vs 6 hrs., $^{*}p$=0.0473; 6 hrs vs 24hrs., $^{*}p$=0.0120. **(e)** PLA assay shows the interation between PHF tau (AT100) and H3K9me3 with UV exposure for 10mins in primary mouse cortical neuron cultures (DIV7). **(f)** Quantification of PLA signals for colocalized p-tau(AT100)-H3K9me3 with DAPI. * *p* = 0.032, n= 5.

Suppl.Fig.5

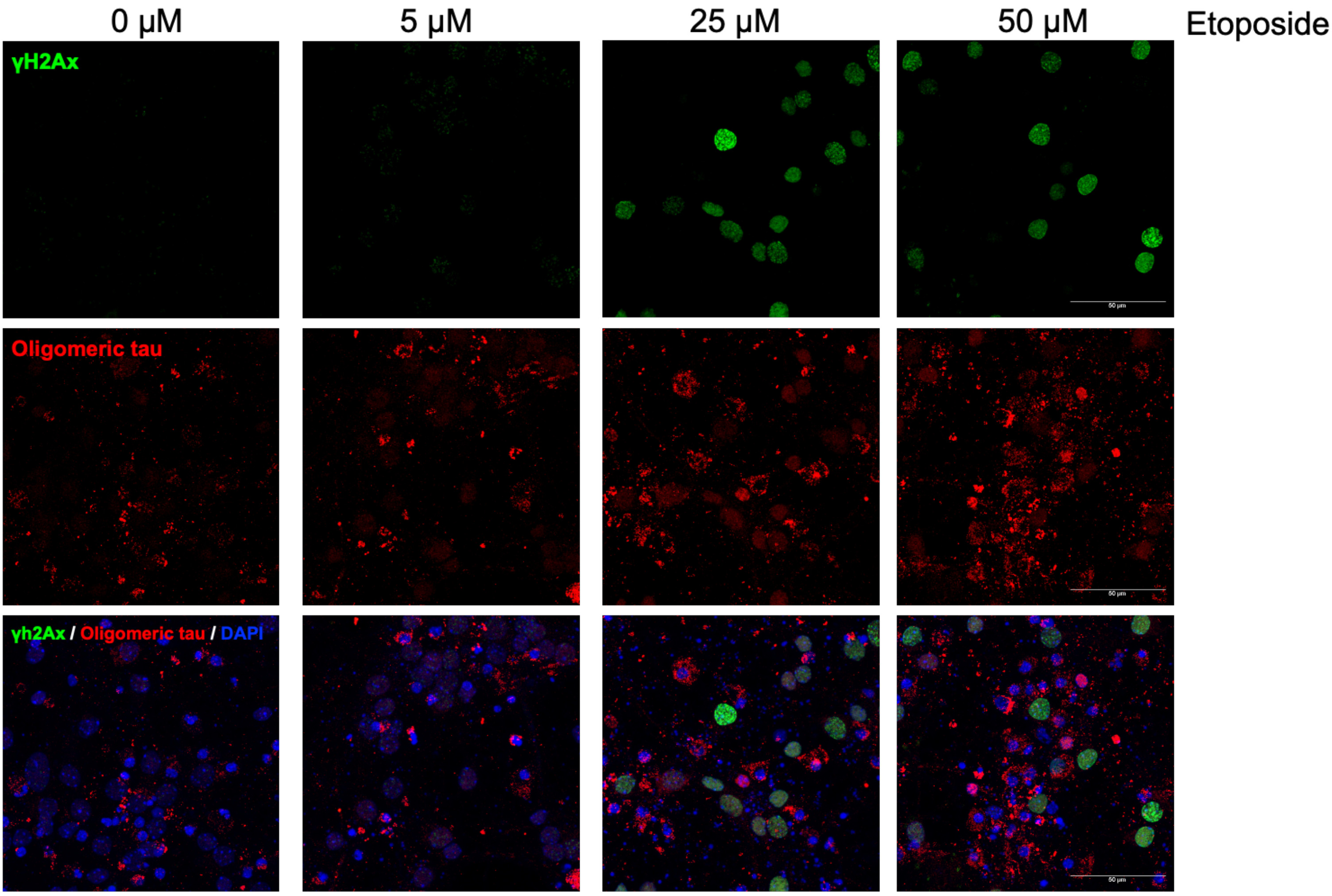

Suppl. Fig. 5 DSB induction by etoposide increase oligomeric tau
Primary mouse cortical neurons (DIV 7) were exposed to 0, 5, 25, and 50 µM etoposide for 24 hrs. Immunofluorescence using antibodies against oligomeric tau (T22, red) and γH2Ax (green). Scale bar=50 µm.

Suppl.Fig.6

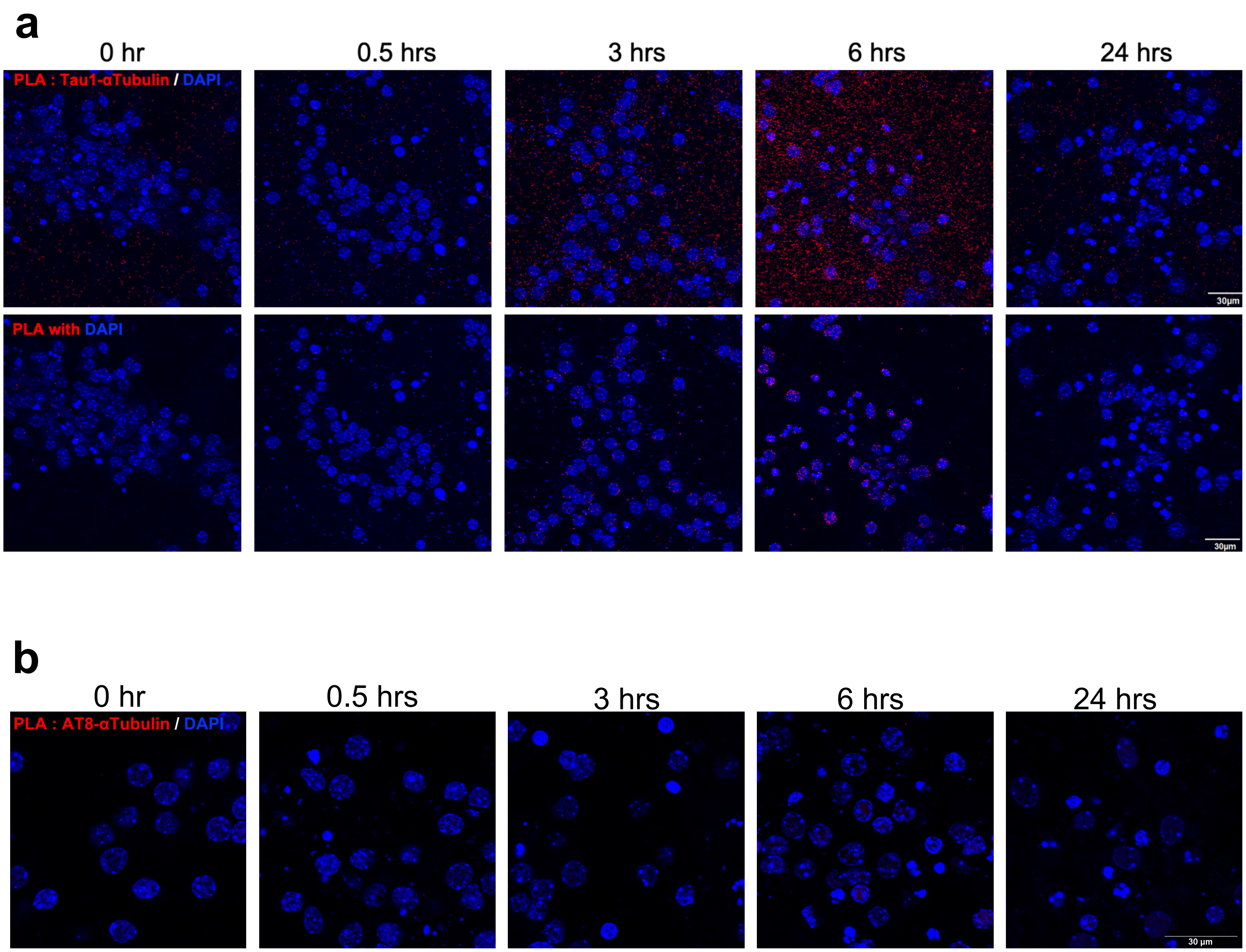

Suppl. Fig. 6 Different co-localization of non-p-tau and non-p-tau with α-tubulin under DSB induction with etoposide

(a) PLA signals (red) against non-p-tau (Tau1) and α-Tubulin. Primary mouse cortical neuron cultures (DIV 7) were treated for 0, 0.5, 3, 6, and 24 hrs with 50 µM etoposide. Bottom panels: PLA foci extraction images of PLA signals with DAPI. Scale bar=30µm. **(b)** PLA signals (red) against p-tau (AT8) and α-Tubulin, under the same same treatment, showing the lack of co-localization. Scale bar=30µm.

Suppl.Fig.7

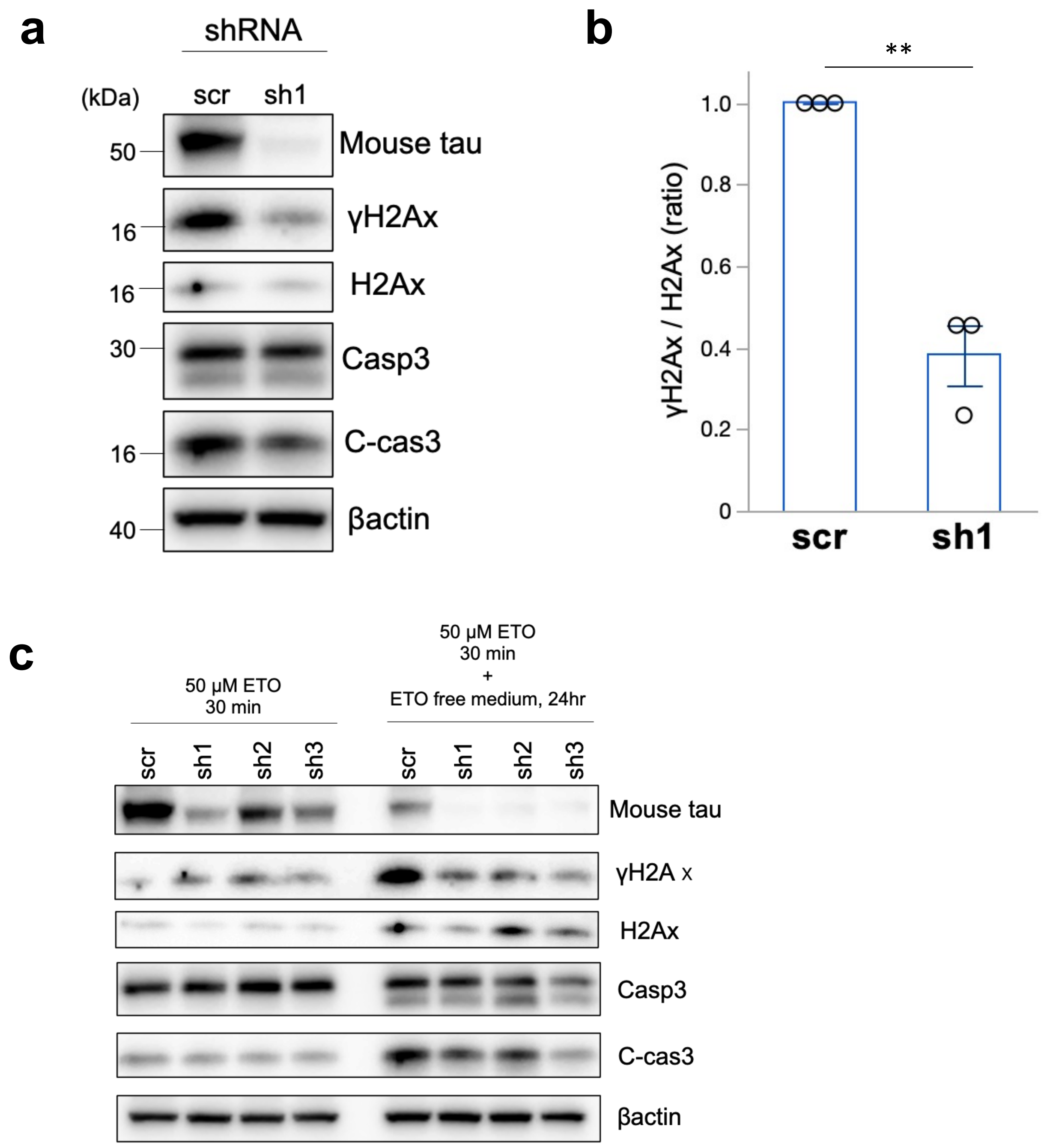

Suppl. Fig. 7 The effect of mouse tau knockdown on DSB accumulation for long term
Primary mouse cortical neuron cultures (DIV 0) were transduced by mouse tau and control shRNA lentivirus particles, cultured for seven days. **(a)** Western bolts of primary neuron samples were treated with 50 μM etoposide for 30 mins, followed by a culture for 24 hrs with an etoposide-free medium. scr, contorol shRNA; sh1, mouse tau shRNA clone 1, Casp3, total caspase 3; C-cas3, cleaved-caspase 3. **(b)** Quantitation of γH2Ax/ H2Ax intensity for Western blot bands, t-test, $^{*}p$=0.002, n=3. **(c)** Western bolts showing the DSB induction, caspase activation by etoposide in primary neuron samples pretreated with three different shRNA against tau. The shRNA-treated neurons were exposed to 50 μM etoposide for 30 mins, subsequently replaced with an etoposide-free medium for another 24 hrs: sh2, mouse tau shRNA clone 2, sh3, mouse tau shRNA clone 3.

Suppl.Fig.8

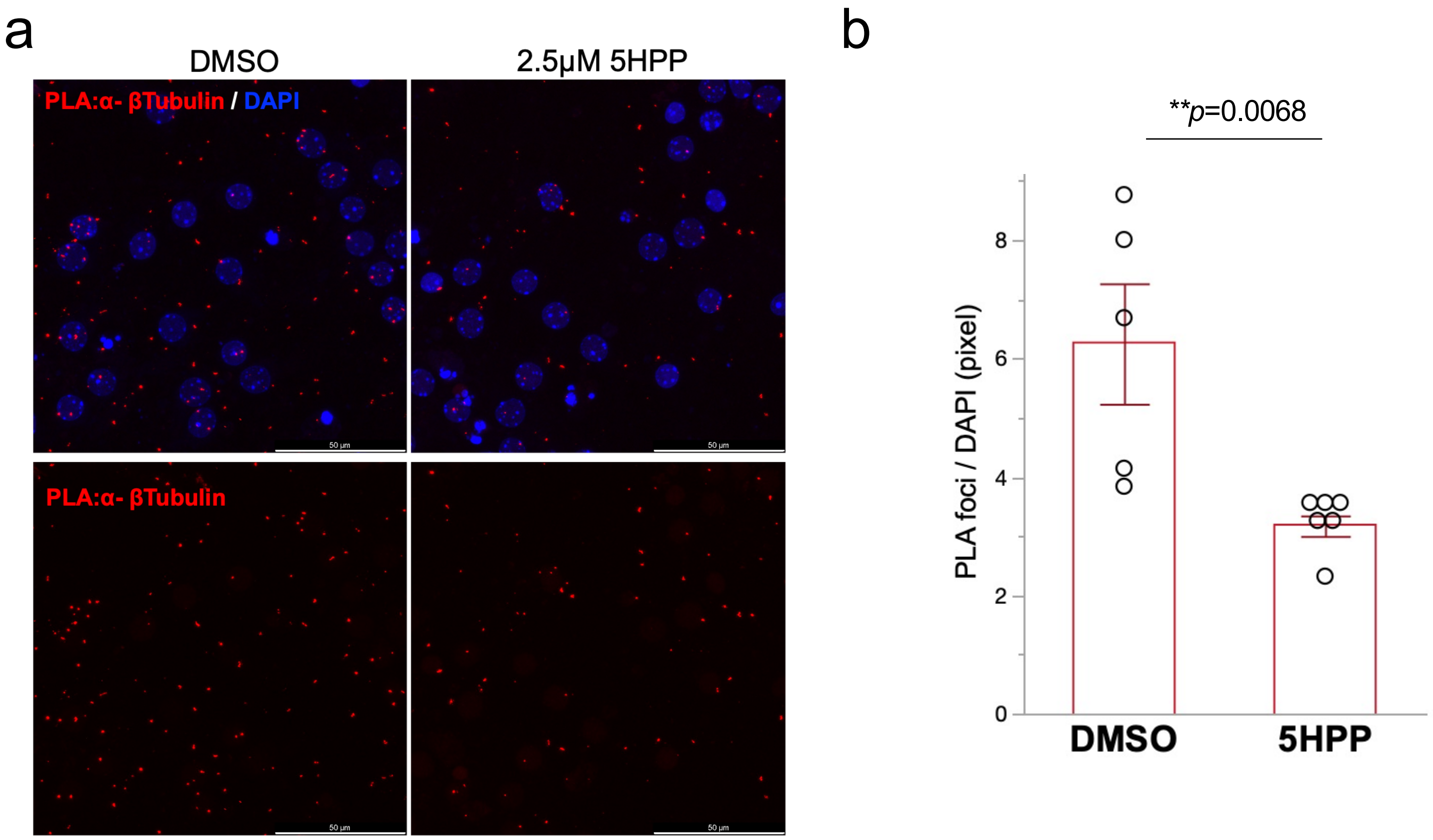

Suppl. Fig. 8 PLA assay for α and β-Tubulin with microtubule polymerization inhibitors
(a) PLA signals (red) for the interaction of α and β-tubulin. DMSO and 2.5 μM 5HPP-33 (microtubule polymerization inhibitor) for 24 hrs in primary mouse cortical neuron cultures (DIV 7). Scale bar=50μm. **(b)** Quantitation of PLA foci per DAPI from primary mouse cortical neurons DMSO and 2.5 μM 5HPP-33 for 24 hrs Determined by one-way ANOVA, followed by a Tukey test. n=6, n: number of images examined; $^{**}p$=0.0068 (Student's t-test).

Suppl.Fig.9

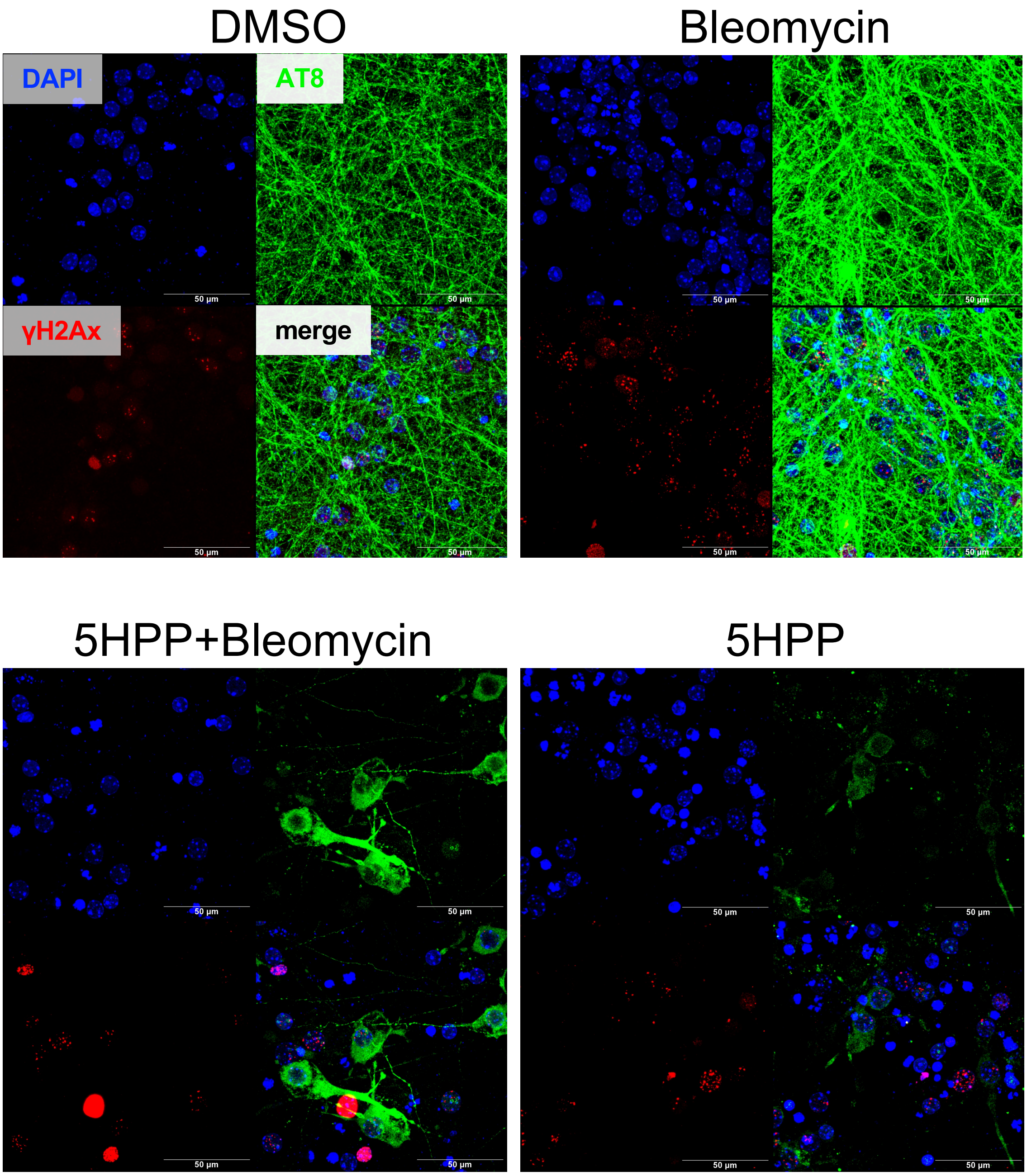

Suppl. Fig. 9 Inhibition of microtubule depolymerization together with DSB induction enhance the cytosolic accumulation of p-tau

Primary mouse cortical neuron cultures (DIV 7) were incubated with DMSO, 10 μg/ml bleomycin, and 10 μM 5HPP-33(5HPP-33: microtubule polymerization inhibitor) for 24 hrs Immunofluorescence was obtained using antibodies against p-tau (AT8, green) and γH2Ax (red). DAPI (blue) was used for labeling the nucleus. Scale bar=50 μm.

Full uncut gels

Megumi Asada-Utsugi et al.

Fig.2 a

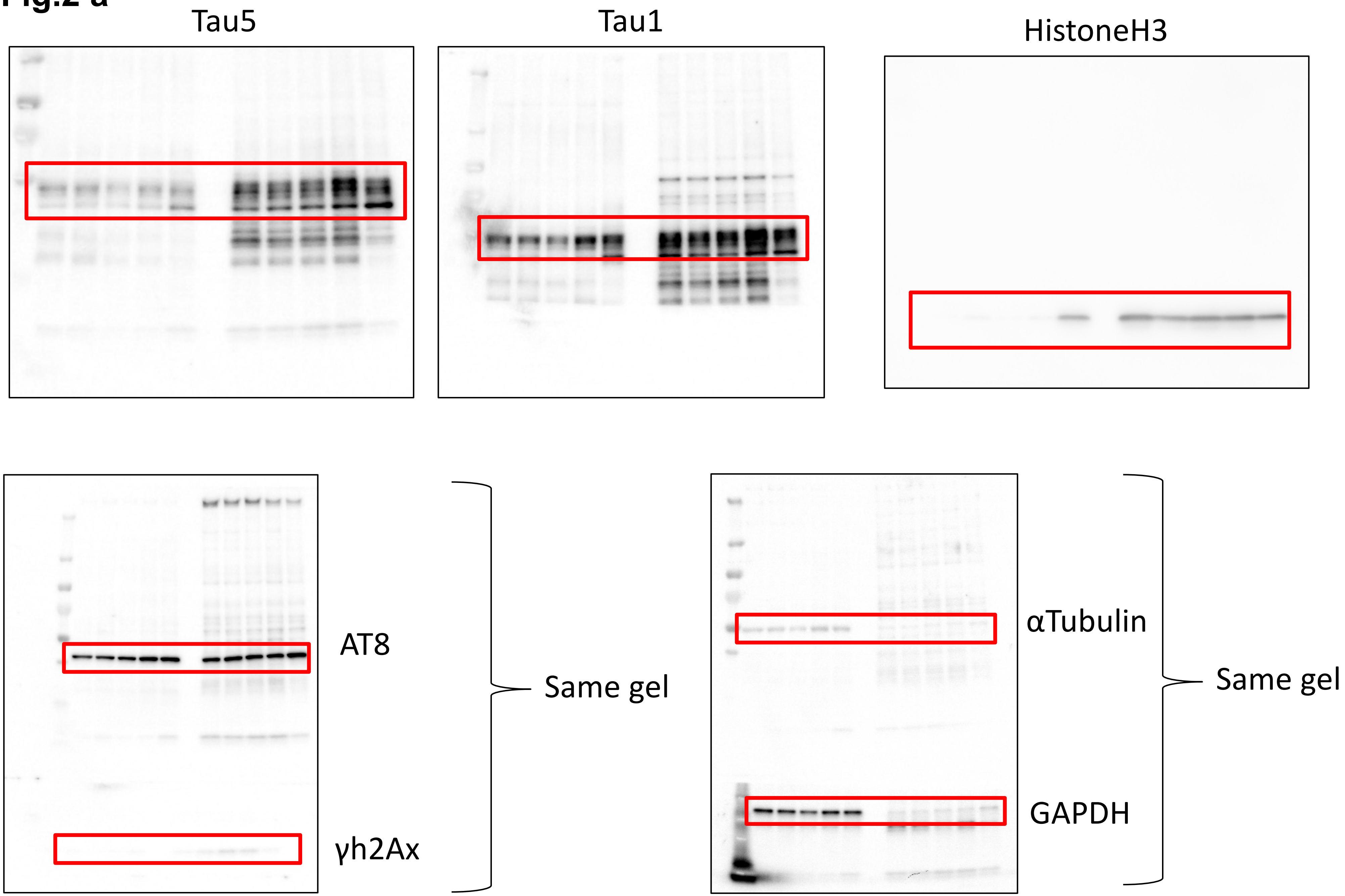
Tau5
Tau1
HistoneH3
AT8
γh2Ax
Same gel
αTubulin
GAPDH
Same gel

Fig.2 a

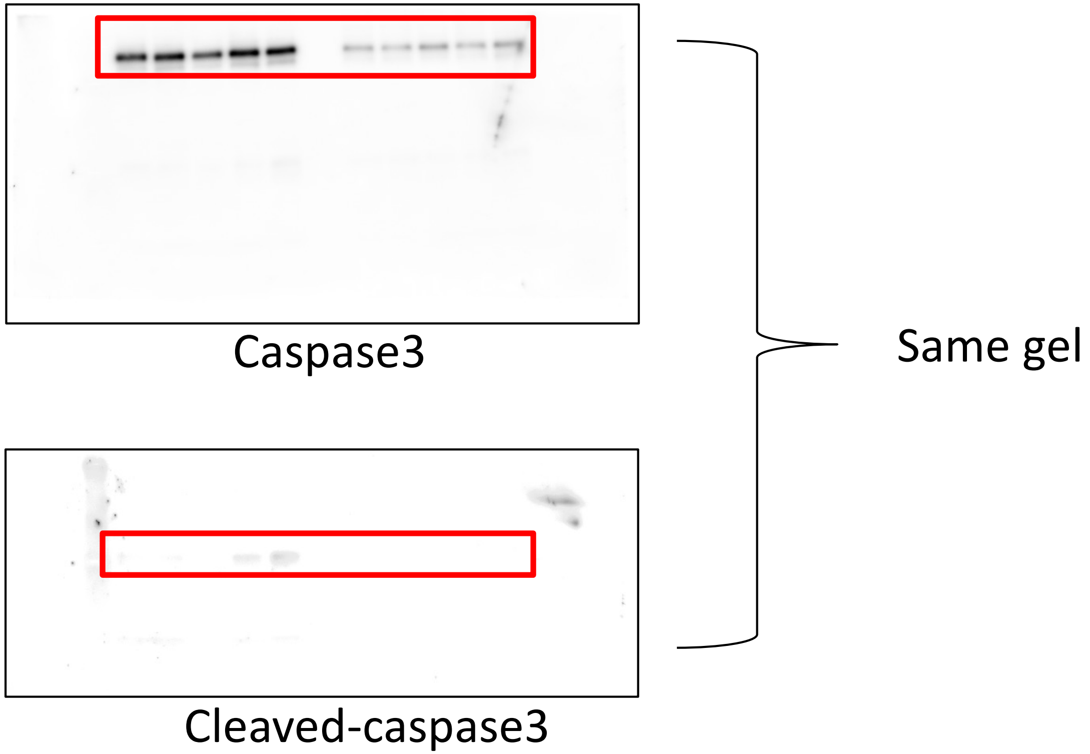

Fig.6 b

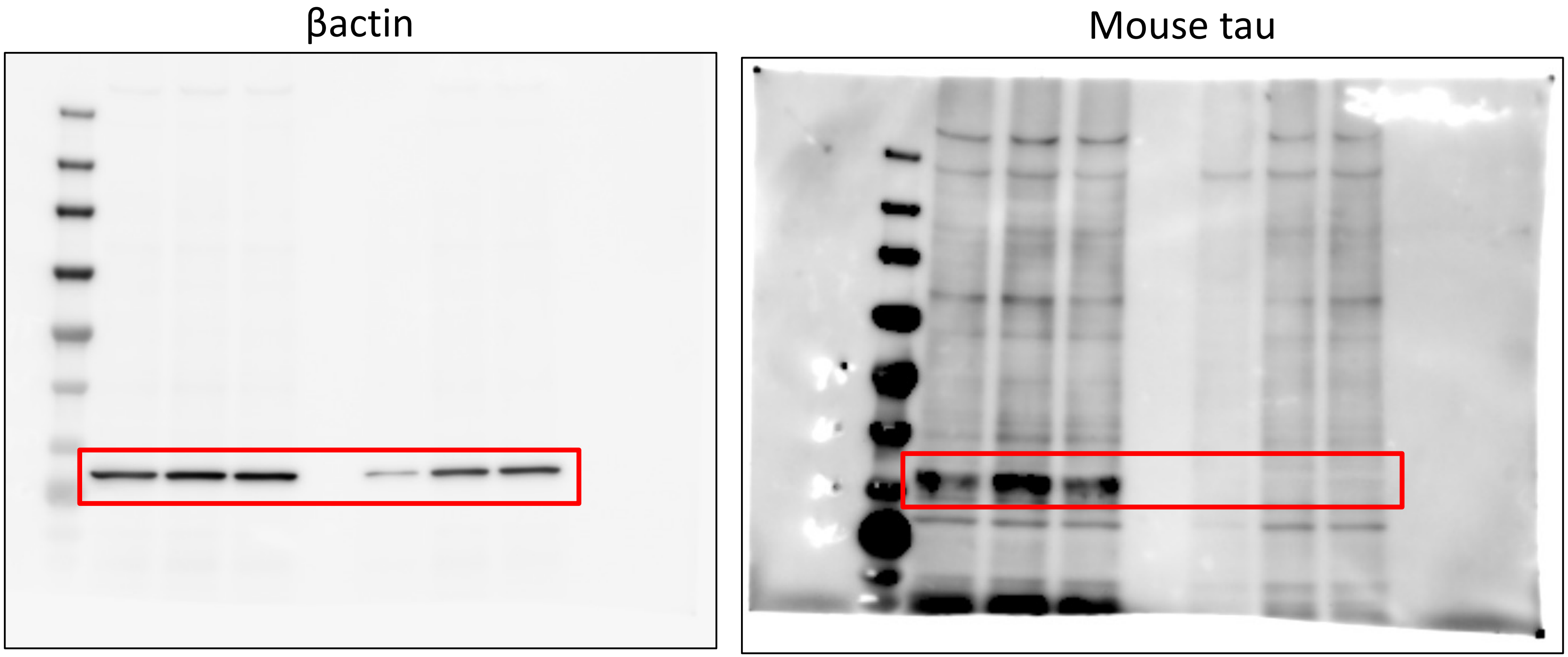

Fig.6 d

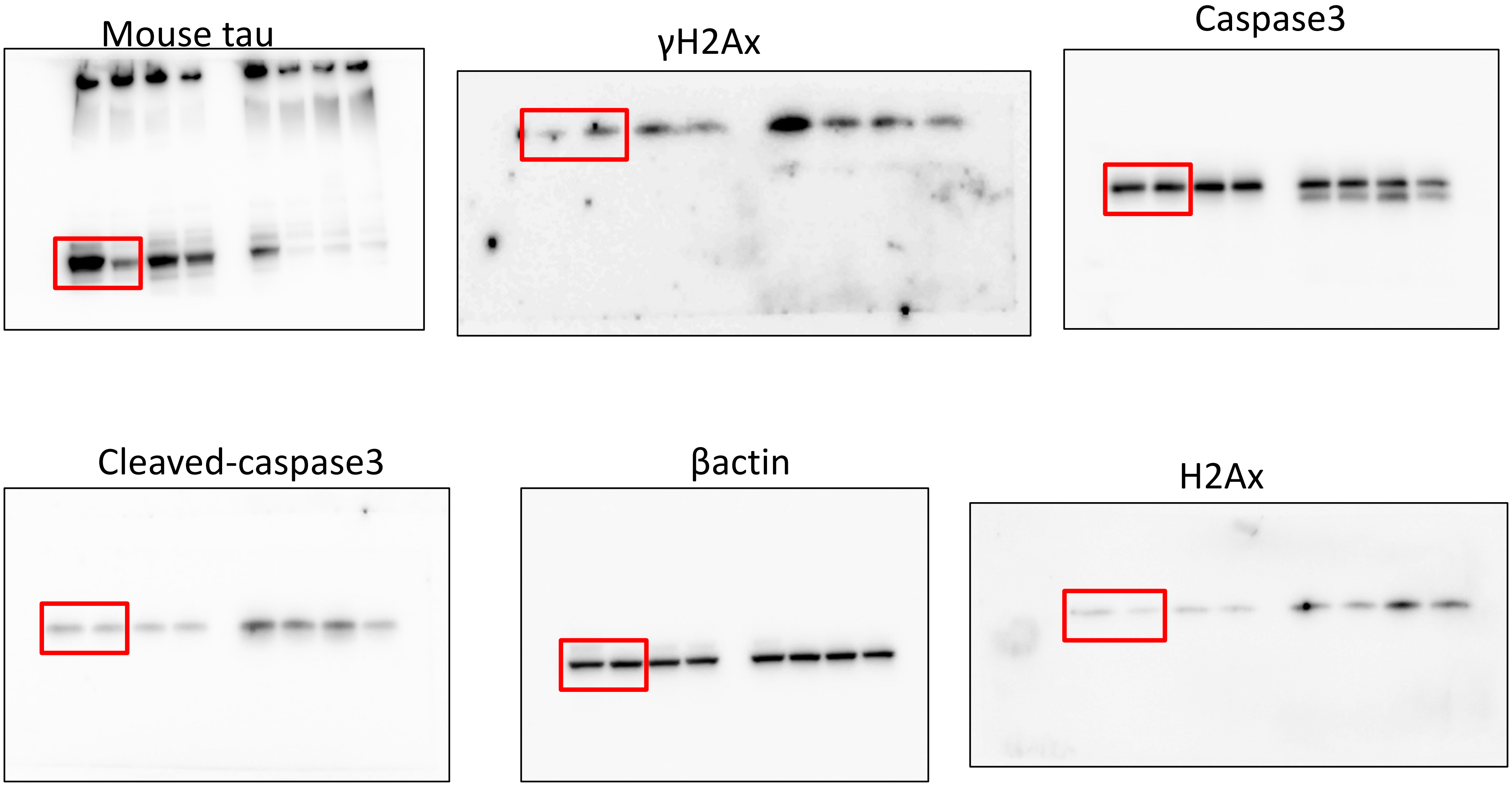

Fig.7 b

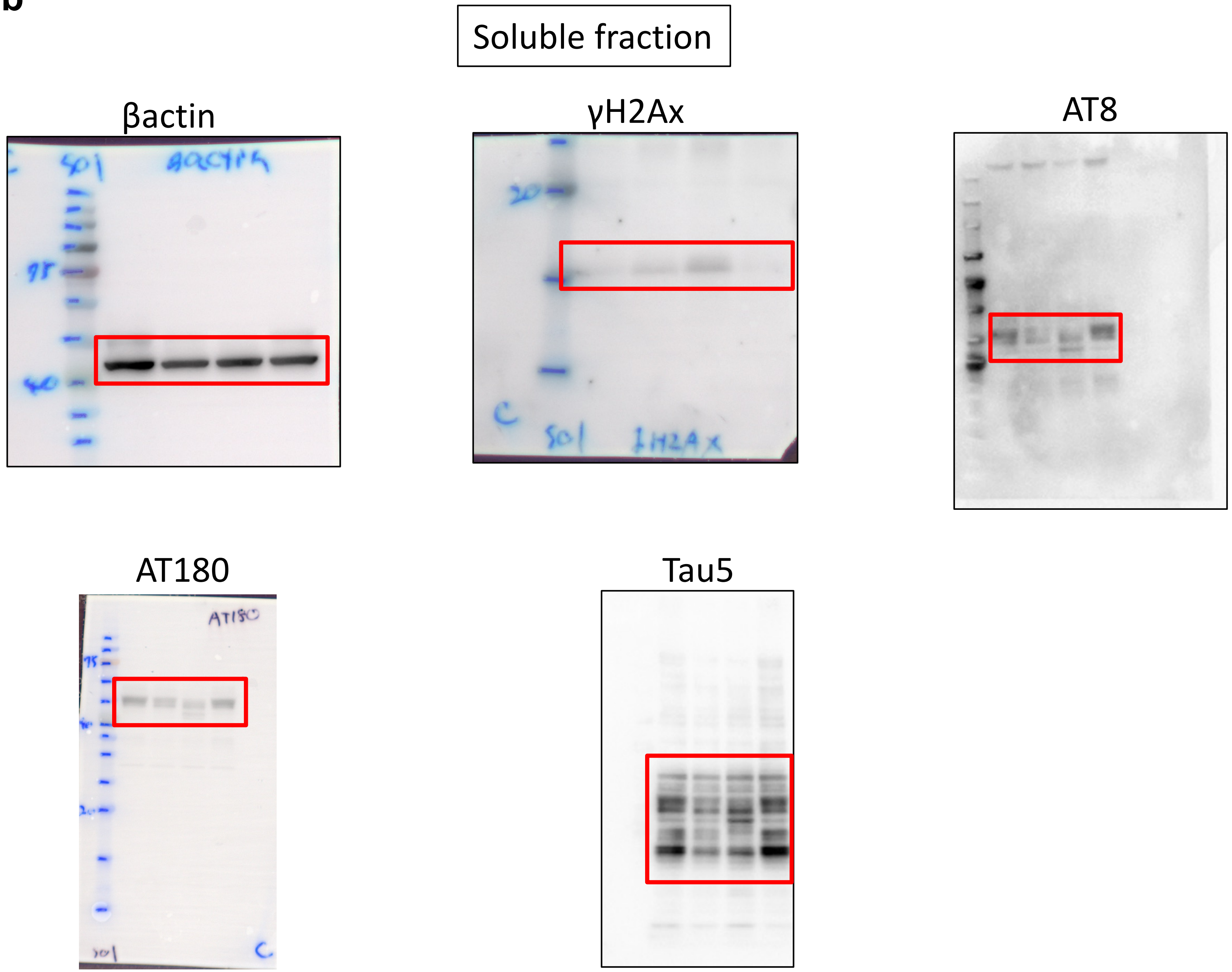

Fig.7 b

Insoluble fraction

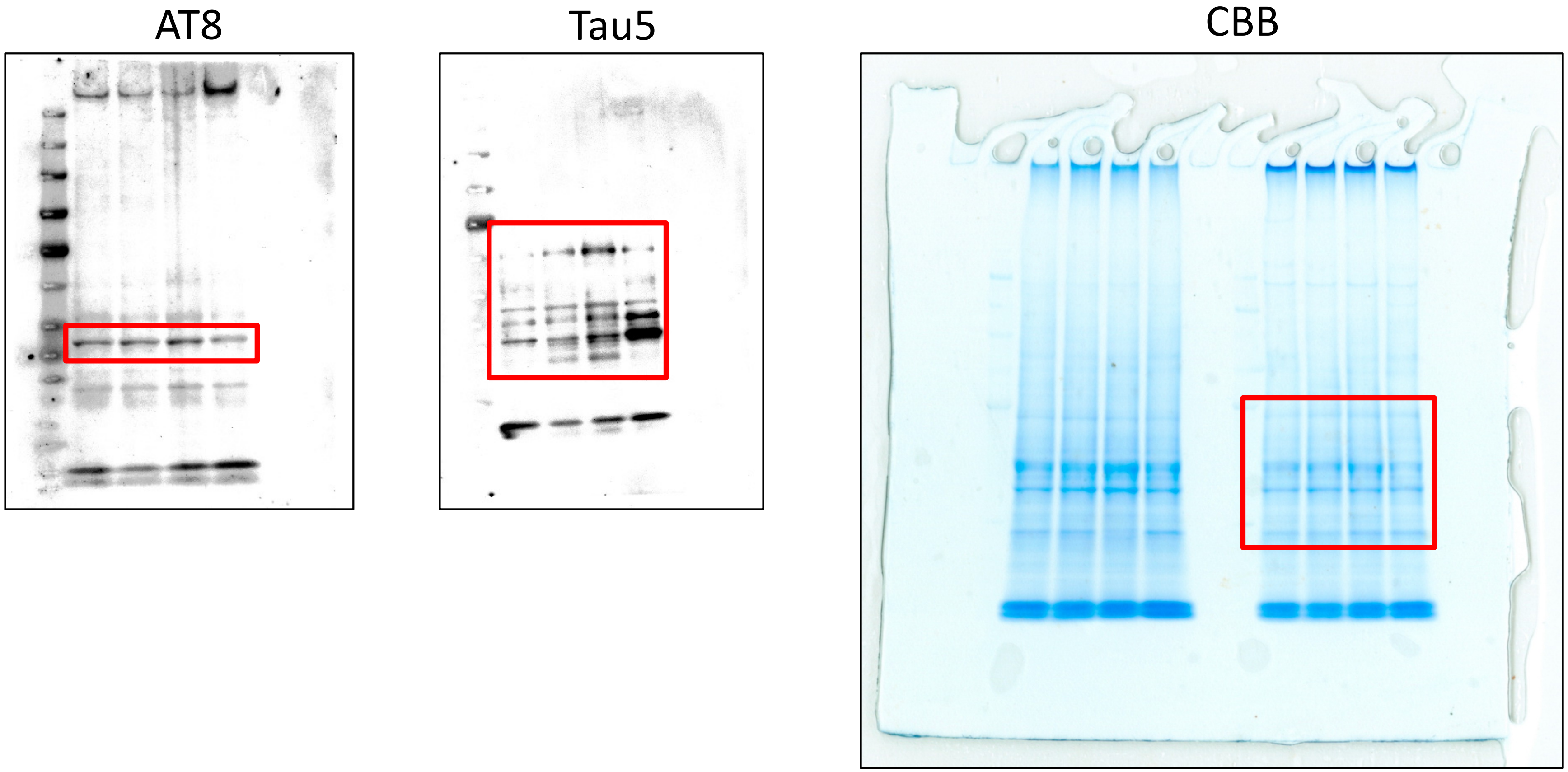

Suppl.Fig.3 a

UV

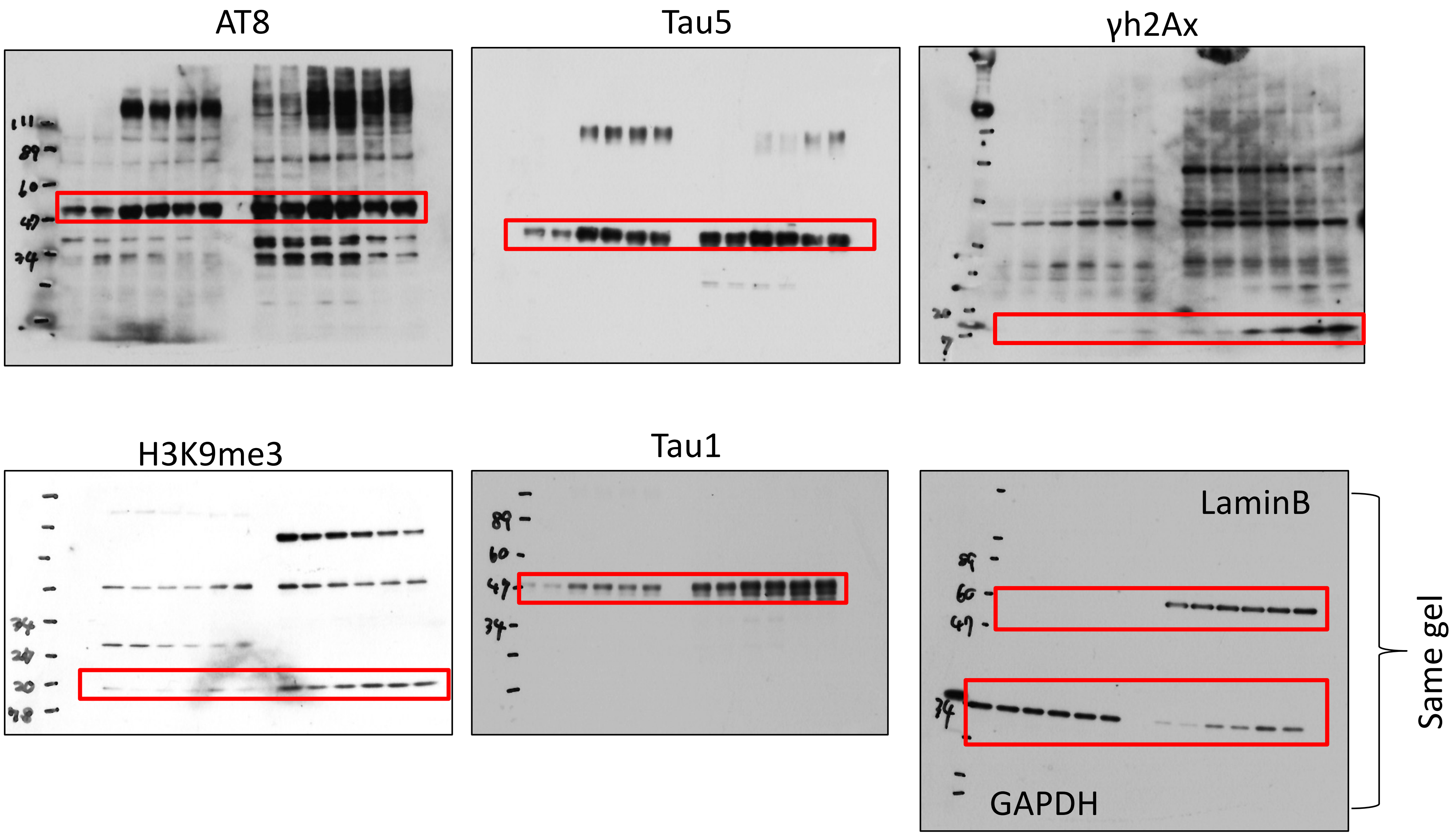

Suppl.Fig.3 b

DIV 14

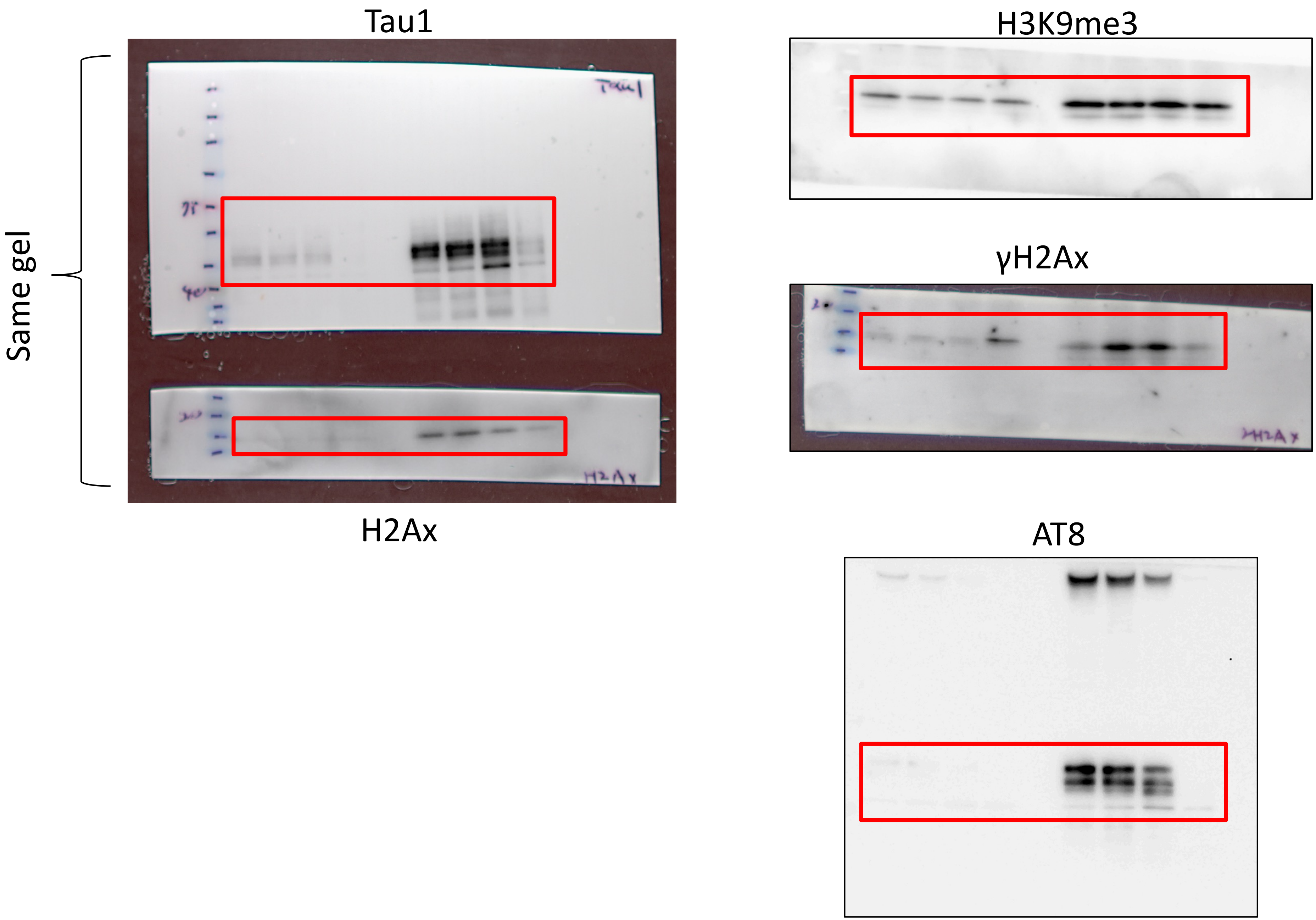

Suppl.Fig.7 a

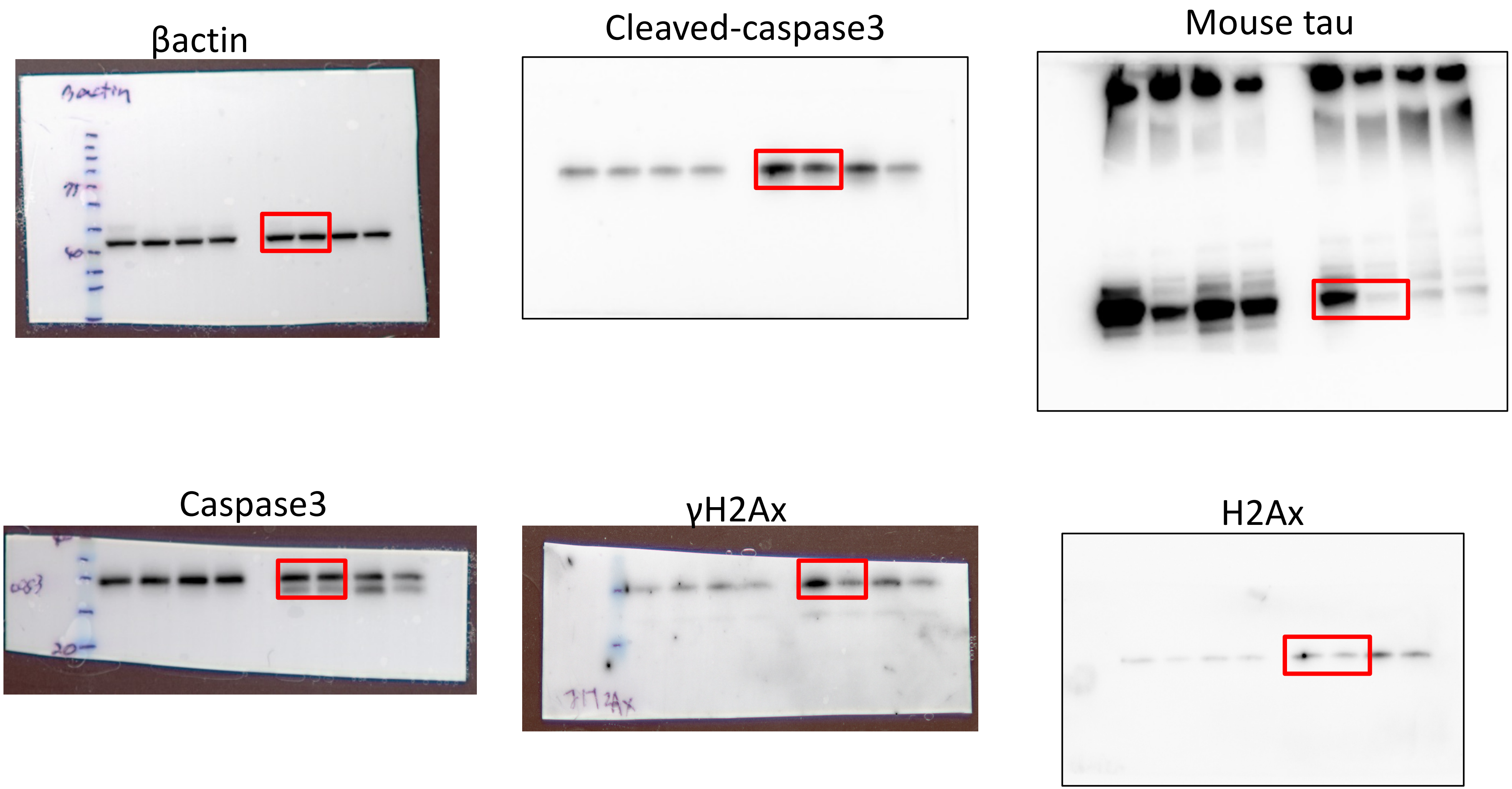

Suppl.Fig.7 c

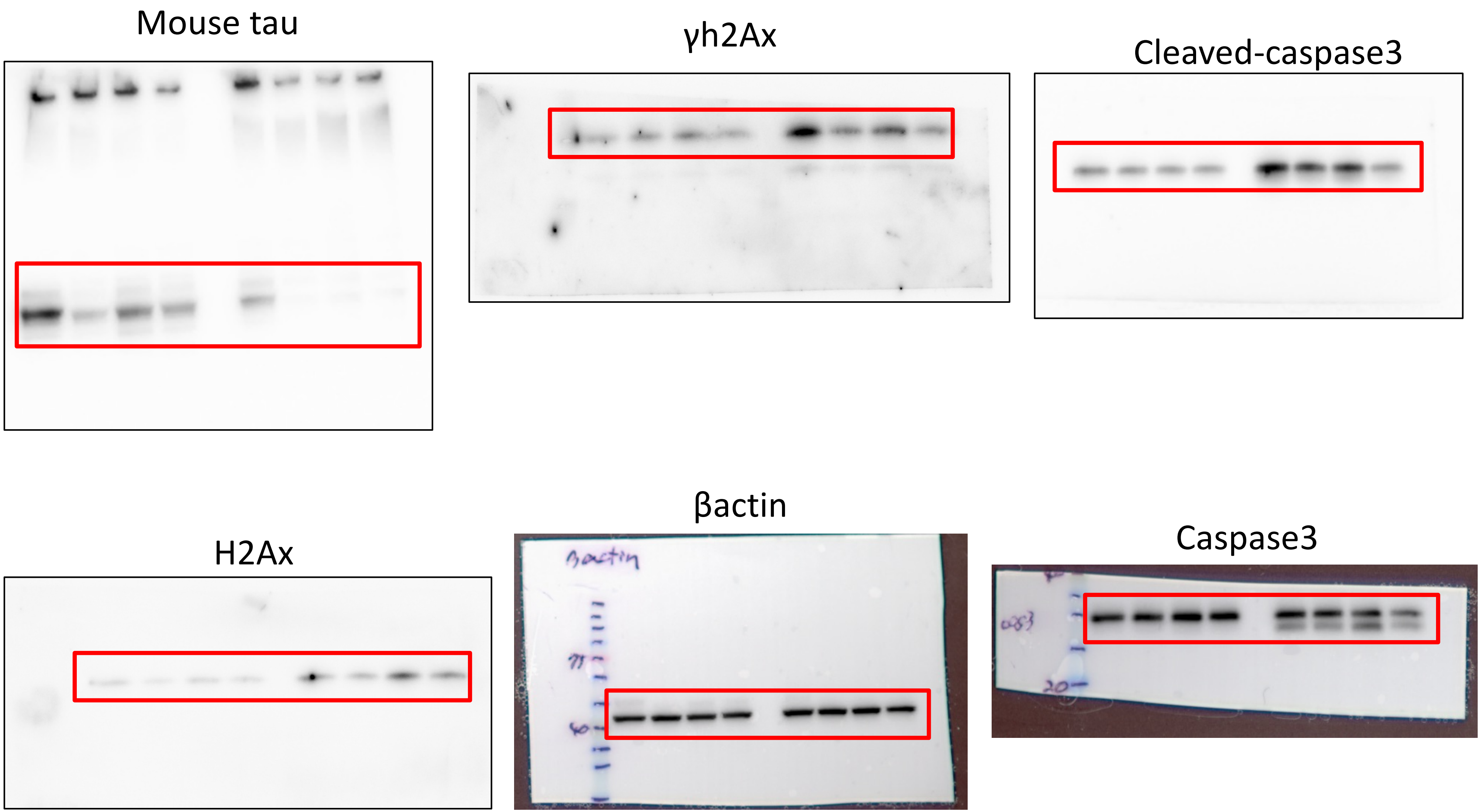

Supplement: Supplementary file 2 — Supplementary information [file 42003_2022_3312_MOESM2_ESM.pdf]
