# Supplementary material for: Failure of DNA double-strand break repair by tau mediates Alzheimer’s disease pathology in vitro
Source: Commun Biol. 2022 Apr 13;5:358. doi: 10.1038/s42003-022-03312-0 (PMC9008043; doi:10.1038/s42003-022-03312-0)
Supplement: Supplementary file 3 — Description of Additional Supplementary Files [file 42003_2022_3312_MOESM3_ESM.pdf]

Description of Additional Supplementary Files

File name: Supplementary Data 1
Description: Quantification raw data for graph presentations.
