# Supplementary material for: Failure of DNA double-strand break repair by tau mediates Alzheimer’s disease pathology in vitro
Source: Commun Biol. 2022 Apr 13;5:358. doi: 10.1038/s42003-022-03312-0 (PMC9008043; doi:10.1038/s42003-022-03312-0)
Supplement: Supplementary file 5 — Reporting Summary [file 42003_2022_3312_MOESM5_ESM.pdf]

nature research

Corresponding author(s): Makoto Urushitani, M.D., Ph.D.

Last updated by author(s): Jan 7, 2022

Reporting Summary

Nature Research wishes to improve the reproducibility of the work that we publish. This form provides structure for consistency and transparency in reporting. For further information on Nature Research policies, see our [Editorial Policies] and the [Editorial Policy Checklist].

Statistics

For all statistical analyses, confirm that the following items are present in the figure legend, table legend, main text, or Methods section.

n/a	Confirmed	
☐	☒	The exact sample size (*n*) for each experimental group/condition, given as a discrete number and unit of measurement
☐	☒	A statement on whether measurements were taken from distinct samples or whether the same sample was measured repeatedly
☐	☒	The statistical test(s) used AND whether they are one- or two-sided<br>*Only common tests should be described solely by name; describe more complex techniques in the Methods section.*
☐	☒	A description of all covariates tested
☐	☒	A description of any assumptions or corrections, such as tests of normality and adjustment for multiple comparisons
☐	☒	A full description of the statistical parameters including central tendency (e.g. means) or other basic estimates (e.g. regression coefficient) AND variation (e.g. standard deviation) or associated estimates of uncertainty (e.g. confidence intervals)
☐	☒	For null hypothesis testing, the test statistic (e.g. *F*, *t*, *r*) with confidence intervals, effect sizes, degrees of freedom and *P* value noted<br>*Give P values as exact values whenever suitable.*
☒	☐	For Bayesian analysis, information on the choice of priors and Markov chain Monte Carlo settings
☒	☐	For hierarchical and complex designs, identification of the appropriate level for tests and full reporting of outcomes
☐	☒	Estimates of effect sizes (e.g. Cohen's *d*, Pearson's *r*), indicating how they were calculated

Our web collection on [statistics for biologists] contains articles on many of the points above.

Software and code

Policy information about [availability of computer code]

Data collection	Not applicable.
Data analysis	Not applicable.

For manuscripts utilizing custom algorithms or software that are central to the research but not yet described in published literature, software must be made available to editors and reviewers. We strongly encourage code deposition in a community repository (e.g. GitHub). See the Nature Research [guidelines for submitting code & software] for further information.

Data

Policy information about [availability of data]

All manuscripts must include a [data availability statement]. This statement should provide the following information, where applicable:

- Accession codes, unique identifiers, or web links for publicly available datasets
- A list of figures that have associated raw data
- A description of any restrictions on data availability

Mouse tau shRNA sequence code is NM_010838.
All the original data can be open and available after the publication.
Raw data have been provided as Excel file as Supplementary data.

Field-specific reporting

Please select the one below that is the best fit for your research. If you are not sure, read the appropriate sections before making your selection.

☒ Life sciences ☐ Behavioural & social sciences ☐ Ecological, evolutionary & environmental sciences

For a reference copy of the document with all sections, see nature.com/documents/nr-reporting-summary-flat.pdf

Life sciences study design

All studies must disclose on these points even when the disclosure is negative.

Sample size	Western blots analysis and mouse tau KD experiments were repeated at least 3 times. PLA assay was repeated 1 or 2 times and analyzed at least 3 images. About human brain slices , AD and control brain slices were used 5 people each on immunohistochemistry.
Data exclusions	Not applicable.
Replication	PLA assay was repeated 1 or 2 times and analyzed at least 3 images. Immunohistochemistry of human brain was repeated 1 or 2 times. All other experiments were done at least three times.
Randomization	Human control brain samples were selected non-neurodegenerative diseases.
Blinding	Human brain slices were blinded by numbering before the experiments.

Reporting for specific materials, systems and methods

We require information from authors about some types of materials, experimental systems and methods used in many studies. Here, indicate whether each material, system or method listed is relevant to your study. If you are not sure if a list item applies to your research, read the appropriate section before selecting a response.

Materials & experimental systems

n/a	Involved in the study	
☐	☒	Antibodies
☒	☐	Eukaryotic cell lines
☒	☐	Palaeontology and archaeology
☒	☐	Animals and other organisms
☐	☒	Human research participants
☐	☒	Clinical data
☒	☐	Dual use research of concern

Methods

n/a	Involved in the study	
☒	☐	ChIP-seq
☒	☐	Flow cytometry
☒	☐	MRI-based neuroimaging

Antibodies

Antibodies used	Aβ (1:500, clone 6E10, BioLegend), γH2Ax (1:1000, #2577), H2Ax (1:1000, #7631), Histone H3 (1:1000, #9717), Caspase3 (1:1000, #9662), cleaved Caspase-3 (1:1000, #9661), α-Tubulin (1:1000, #2144, Cell Signaling Technology, MA), NeuN (1:500, EPR12763, abcam, UK), MAP2 (1:1000, clone Ap20, BD Biosciences, NJ), AT8 (1:1000, MN1020), AT180 (1:1000, MN1040), AT100 (1:1000, MN1060), ZO-1 (1:500, 40-2200), Tau5 (1:1000, AHB0042, Thermo Fisher Scientific, MA), γH2Ax (1:1000, host mouse, clone JBW301,#05-636), Tau-1 (1:1000, clone PC1C6, MAB3420), Olig2 (1:500, AB9610), β-actin (1:10000, A5441), H3K9me3 (1:1000, 05-499), Tau oligomeric (T22, 1:1000,#ABN454, millipore, CA), LaminB (1:1000, M-20, sc-6217), β Tubulin (1:1000, sc-5274), GAPDH (1:5000, FL-335, sc-25778, santa cruz biotech.), GFAP (1:500, G 3893, Merck, DE), mouse tau (1:1000, 012-26963) and Iba1 (1:500, 013-27691, FUJIFILM Wako Chemical Coporation, JP); mouse, rabbit and goat IgG HRP-conjugated (Jackson ImmunoResearch, PA).
Validation	Many antibodies were used for WB, immunohistochemistry and immunocytometory . Mouse, rabbit and goat IgG HRP-conjugated (Jackson ImmunoResearch, PA) were used for 2nd anti-body in western blots.

Human research participants

Policy information about studies involving human research participants

Population characteristics	Human data was obtained from autopasy specimen with antemortem approval of patients or guardians.
Recruitment	The same as above

Ethics oversight	Every research was carefully scrutinized and surveyed by ethical commitee of Shiga University of Medical Science and Kyoto University

Note that full information on the approval of the study protocol must also be provided in the manuscript.

Clinical data

Policy information about clinical studies

All manuscripts should comply with the ICMJE guidelines for publication of clinical research and a completed CONSORT checklist must be included with all submissions.

Clinical trial registration	'Pathological and biochemical studies of neurodegenerative diseases using human autopsy brain and spinal cord' (No. R1038) by the Kyoto University Ethics Committee.
Study protocol	This research is not a clinical studies, and comprehensive permitting system was utilized as to use autopsy specimen in Shiga Univiersity of Medical Science and Kyoto University.
Data collection	Not applicable (This study presents only immunohistochemistry for research, which does not affect the diagnosis or treatment)
Outcomes	Not applicable.
